# Supplementary material for: Dasatinib and Quercetin Alleviate Retinal Ganglion Cell Dendritic Shrinkage and Promote Axonal Regeneration in Mice with Optic Nerve Injury
Source: Int J Mol Sci. 2025 Dec 18;26(24):12170. doi: 10.3390/ijms262412170 (PMC12733733; doi:10.3390/ijms262412170)
Supplement: Supplementary file 1 [file ijms-26-12170-s001.zip › ijms-3976034-supplementary.pdf]

Supplementary Table S1: Primers for gene expression analysis.

Genes		Sequence (5' > 3')	Annealing Temp (ºC)	Amplicon size (bp)	References
Rpl19	F:	TGAAATCGCCAATGCCAACTC	60	195	NM_009078.2
	R:	CAGGTCACCTTCTCAGGCAT			
Pdc	F:	GACCCAAACTACTACACCAAACC	60	178	NM_024458.2
	R:	TTCCTTCTTGCTGGGTGGAAT			
Nrtn	F:	TGGTCGGAGGAGTCACCA	60	104	NM_008738.4
	R:	CTGCAGATGAGCGACACCA			
Metrn	F:	CGAGGACCAACGTGCAGAAA	60	128	NM_133719.2
	R:	TGGTCCCGTGGATCACAAAATC			
Tma7	F:	CCCCTGAAACAGCCCAAGAA	60	187	NM_183250.2
	R:	CACCCCAGATGTGAGGAACA			
Fstl5	F:	AAGAAGCCGTTGGTGGATCA	60	172	NM_178673.4
	R:	GGTGCTTGTCAGCGTTGAAG			
Uba6	F:	GGTGCAGGGAGTCAAAATGC	60	163	NM_172712.2
	R:	GCCGGGCAAATCTTCATCTC			
Slc38a2	F:	GCGTATGGTCTGGCTGGAAA	60	196	NM_175121.4
	R:	TGAGGACAAGGGACACCAGA			
Zfhx4	F:	TGAACCCTCTCGTTTTACTTGGA	60	193	NM_030708.2
	R:	TGCCCATTTTCCTGCCTTGA			
Ryr3	F:	GAGACAGCAAAAGCAAGATGTCA	60	121	NM_001319156.1
	R:	GCCACGATAAGGGACGTCTG			
Pcsk1	F:	CTATCAGCAGTGCCTCCCAG	60	135	NM_013628.3
	R:	TGCAGTCATTGTGCAGGTCA			
Fosl2	F:	CCAATCCCTATCCACGCTCA	60	172	NM_008037.4
	R:	CGGATTCGACGCTTCTCCT			
Fat4	F:	CATTGCTGGTTCGTGCAGAC	60	158	NM_183221.4
	R:	AATAGCTGCCACCAGGGAAC			
Papolb	F:	CCCAAGAGTGAATCCTAGTGACA	60	112	NM_019943.2
	R:	AATCATGACCATCCTGGTCGAAA			
Mthfd2	F:	ATGGCTTCAGTTTCCTTGTTGTC	60	139	NM_008638.2
	R:	GGGCCAGTTTCCTTCCAGAAA			
Actb	F:	GCTCCGGCATGTGCAAAG	60	100	NM_007393.5
	R:	CCCACCATCACACCCTGG			
